# Identification of risk factors of developing pressure injuries among immobile patient, and a risk prediction model establishment: A protocol for systematic review

**DOI:** 10.1097/MD.0000000000023640

**Published:** 2020-12-24

**Authors:** Ke-Lu Yang, Lin Chen, Ying-Ying Kang, Li-Na Xing, Hai-Ling Li, Peng Cheng, Zong-Hui Song

**Affiliations:** a Evidence-Based Nursing Center, School of Nursing, Lanzhou University; b Gansu trauma Orthopedic hospital; c School of Basic Medical Sciences, Lanzhou University, Lanzhou; d Inner Mongolia Medical University, Hohhot; e Department of Orthopaedics, the Second Hospital of Lanzhou University; f Affiliated Hospital of Gansu University of Chinese Medicine, Lanzhou, China.

**Keywords:** clinical practice guidelines, Pressure injury, risk prediction model, systematic reviews

## Abstract

**Backgroud:**

Pressure injuries (PIs) bring a considerable physical and mental burden on immobile patients, and have put families and government under tremendous pressure to cover the cost of treatment. Therefore, this protocol proposes to identify risk factors of developing PIs in immobile patients from systematic reviews (SRs) and clinical practice guidelines (CPGs), in order to establish a risk prediction model for developing PIs and identify individual risk factors that can be modified to aid prevention.

**Methods:**

Electronic databases and specific databases for CPGs and SRs will be searched. Study selection and data collection will be performed independently by two reviewers. All included SRs and CPGs will be subject to critical appraisal. RevMan 5.3 will be used to calculate the pooled odds ratio (ORP) after appraising the quality of eligible studies, and the risk predictive model will be established using logistic regression model. A narrative synthesis, evidence summary table, and Sankey diagram will also be performed.

**Results:**

The results of this study will be submitted to a peer-reviewed journal for publication.

**Conclusion:**

This systematic review will provide a risk prediction model of PI developing.

**INSPLAY registration number:**

INPLASY2020100097

## 1 Introduction

Pressure injuries (PIs) is localized damage to the skin and underlying soft tissue usually over a bony prominence or related to the medical device, and it considerably threatens the health of humans due to high incidence rate and severe complications.^[1,2]^ The global prevalence of PIs in hospitalized adult patients has reached at 12.8%, especially in low-income countries, such as Ethiopia, the prevalence of PIs in a Ethiopia hospital was up to 14.9%.^[3,4]^ Besides, PIs represents a significant medical burden with €2.5 billion spent annually and €1.71 to €470.49 per patient on PIs treatments in Europe.^[1,5,6]^ In addition to bear such heavy medical expenses, patients with PIs may suffer considerable pain and discomfort associated with wounds, so PIs has been seen as an outcome of poor-quality nursing care.^[7,8]^

Particularly, immobile patients seem to be at a higher risk of developing PIs.^[9,10]^ Meanwhile, stroke, the leading cause of paralysis, is the third most common cause of death globally and is shown a growing incidence among young adult patients, which may increase the number of immobile patients.^[11–13]^ PIs prevention, considered as the most efficient method of dealing with PIs problems, is very complex, involving multiple interventions and processes and comprising numerous interacting components, and assessment of risk factors is the primary area of prevention measures.^[14–16]^ Risk assessment is a central component of clinical practice aimed at identifying individuals susceptible to PIs in order to target appropriate interventions and prevent PIs development.^[2,17]^ Degrees of mobility, perfusion, and skin status have been identified as the most common independent risk factors for the development of PIs.^[18]^ Moreover, factors such as pain, urination problems, nutritional and general health status have been associated with PIs risk.^[17,19]^ Nevertheless, no single factor can explain PIs risk, instead, there is a complex interplay of factors that increase the probability of PIs development.^[20]^ An improved understanding of the relative contribution risk factors and an enhanced capacity of identifying immobile patients about to develop PIs would enable us to reduce the incidence of PIs and better target resources in practice.^[21]^ However, agreement on the predictive risk factors is lacking has led to the propagation of different tools, such as Braden, Norton, and Waterlow scales, which have all shown low sensitivity and specificity in identifying at-risk patients.^[17,22,23]^

Clinical practice guidelines (CPGs), considered as the highest level evidence, has summarized common risk factors of PIs development. Meanwhile, many systematic reviews (SRs) on identifying risk factors of developing PIs have been published internationally.^[24,25]^ Therefore, the aim of this study is to identify risk factors for the development of PIs in immobile patients based on SRs and CPGs, and establish a risk prediction model to predict the probability of occurrence of PIs development for immobile patients based on the risk factors we collected.

## 2 Methods and analysis

### 2.1 Protocol and registration

This study will be reported according to the Preferred Reporting Items for Systematic Reviews and Meta-Analyses (PRISMA-P) and the checklist is presented in online Supplementary Appendix 1. The systematic review was registered in the I International Platform of Registered Systematic Review and Meta-analysis Protocols (INSPLAY) database (protocol number: INPLASY2020100097). Ethical approval is not required because this is a literature-based study.

### 2.2 Search strategy

Search strategies will be performed on the following electronic databases: PubMed, EMABSE, Web of Science, CINAHL Complete, and Cochrane Library. Specific database for CPGs will be searched, for example: The National Institute for Health and Care Excellence (NICE) (www.nice.org.uk), Scottish Intercollegiate Guidelines Network (SIGN) (https://www.sign.ac.uk/), National Pressure Injuries Advisory Panel (NPIAP) (https://npuap.org/), RNAO (https://communities.rnao.ca/), European Pressure Ulcer Advisory Panel (EPUAP) (http://www.epuap.org/), and so on. The MeSH search and text word search will be used with the terms related to pressure injury, pressure ulcer, decubitus ulcer, and risk factors. The specific search strategy will be (taking PubMed as an example) is shown in Table 1. The strategy will be modified for other databases use if necessary.

**Table 1:** Searching strategy in PubMed.

#1	“Pressure Ulcer”[Mesh] OR pressure ulcer[Title/Abstract] OR pressure ulcers[Title/Abstract] OR PU[Title/Abstract] OR decubus ulcer[Title/Abstract] OR bedsore[Title/Abstract] OR bedsores[Title/Abstract] OR pressure sore[Title/Abstract] OR pressure sores[Title/Abstract] OR pressure injury[Title/Abstract] OR PI[Title/Abstract] OR bed sore[Title/Abstract] OR decubitus ulcer[Title/Abstract] OR decubitus ulcers[Title/Abstract] OR decubital ulcer[Title/Abstract] OR decubital ulcus[Title/Abstract] OR decubitus ulceration[Title/Abstract] OR decubitus ulcus[Title/Abstract] OR decubus ulcer[Title/Abstract] OR ulcus decubitus[Title/Abstract]
#2	“Guideline” [Publication Type] OR “Guidelines as Topic”[Mesh] OR “Practice Guideline” [Publication Type] OR “Critical Pathways”[Mesh] OR “Algorithms”[Mesh] OR “Health Planning Guidelines”[Mesh] OR “Consensus”[Mesh] OR guideline[Title/Abstract] OR guidance[Title/Abstract] OR standard[Title/Abstract] OR critical pathways[Title/Abstract] OR practice guidelines[Title/Abstract] OR clinical practice guidelines[Title/Abstract] OR quality[Title/Abstract] OR best practice[Title/Abstract] OR health planning guidelines[Title/Abstract] OR recommendation[Title/Abstract] OR consensus[Title/Abstract]
#3	“Meta-Analysis” [Publication Type] OR “Meta-Analysis as Topic”[Mesh] OR “Systematic Reviews as Topic”[Mesh] OR “Systematic Review” [Publication Type] OR systematic review[Title/Abstract] OR systematic reviews[Title/Abstract] OR systematic study[Title/Abstract] OR systematic studies[Title/Abstract] OR meta analysis[Title/Abstract] OR meta analyses[Title/Abstract] OR metaanalysis[Title/Abstract] OR metanalysis[Title/Abstract] OR met-analysis[Title/Abstract] OR metaanalyses[Title/Abstract] OR metanalyses[Title/Abstract] OR met-analyses[Title/Abstract] OR OR meta-study[Title/Abstract] OR meta study[Title/Abstract] OR meta-studies[Title/Abstract] OR meta studies[Title/Abstract]
#4	#1 AND #2
#5	#1 AND #3

The reference lists of eligible studies will be checked by reviewers in order to identify other possible guidelines. For guidelines published only in summary or where important information is missing, we will try to search for complete information by contacting the authors.

### 2.3 Eligibility criteria

#### 2.3.1 Inclusion criteria

We will include the latest version of CPGs and SRs, which aim to identify the risk factors associated with PIs development in immobile adult patients or patients who are unable to reposition without assistance at least 24 hours. Reasons for being immobile include: under sedation, disease-related immobility, and bed rest requirements for disease treatment. Any stage of PIs and any setting will be covered without restriction. There is no time limitation, and language is restricted to English and Chinese.

#### 2.3.2 Exclusion criteria

The summary of the CPGs; the translation of a CPG published in another language; consensus, evidence summary and potocols will be excluded. Duplicate publication of the patient dataset will be excluded.

### 2.4 Study selection

**Figure 1:**
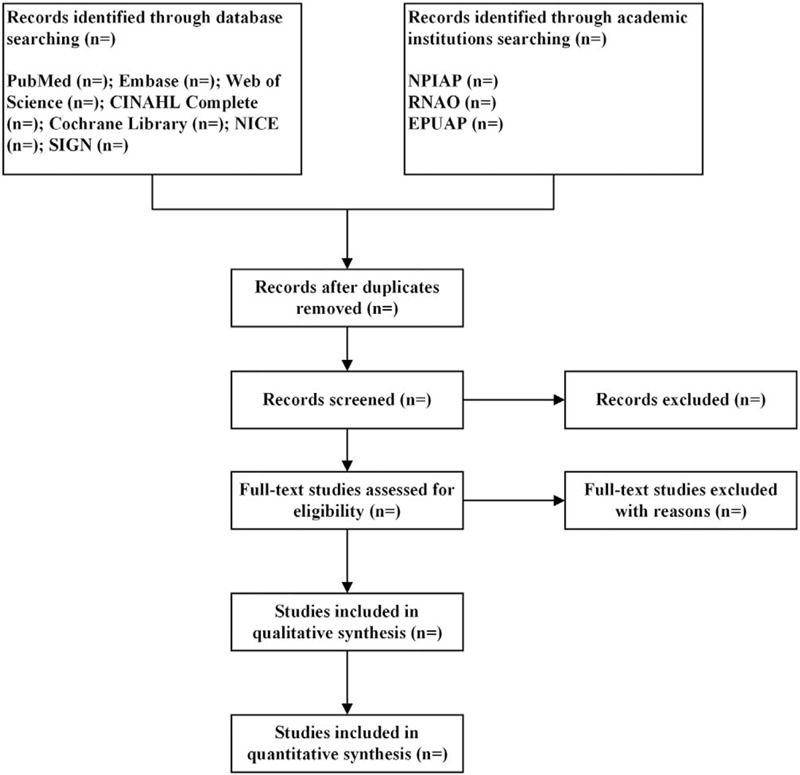
Flow diagram of literature screened. CINAHL = Cumulative Index to Nursing and Allied Health Literature, EPUAP = European Pressure Ulcer Advisory Panel, NICE = National Institute for Health and Care Excellence, NPIAP = National Pressure Injuries Advisory Panel, RNAO = Registered Nurses Association of Ontario, SIGN = Scottish Intercollegiate Guidelines Network.

Literature search records will be imported into EndNote X8 literature management software (Thomson Reuters [Scientific] LLC, Philadelphia, PA). Two reviewers will screen the titles and abstracts of retrieved studies to identify potentially eligible studies. Then they will select the full-text of potentially eligible studies and determine study according to inclusion or exclusion. All the works above will be done independently. Any disagreement will be resolved by the third reviewer. The selection process will be summarized according to PRISMA flow diagram (Fig. 1).

### 2.5 Data extraction

Firstly, a predesigned data extraction form is to be designed by our team. Then, one to five included studies will be pre-extracted. If necessary, the forms shall be continually modified until the final data extraction form complete. Two reviewers will independently extract data from included CPGs and SRs. Different opinions will be resolved through discussion or consult the third party.

Primarily, we will select the candidate risk factors from the latest CPGs, and the following items will be extracted from SRs:

(1) General characteristics: number of authors, year of publication, organizations, or others;
(2) Specific characteristics: stage of PIs, target population, type of studies included in systematic reviews, or others;
(3) Different type of risk factors;
(4) Summary effect sizes and significance levels will be extracted if available.

### 2.6 Assessment of methodological quality

The methodological quality of each of the included systematic reviews and meta-analyses will be assessed independently by two reviewers using A Measurement Tool to Assess Systematic Reviews 2 (AMSTAR 2).^[26,27]^ The overall confidence of a review article in 4 categories: High (no or one non-critical weakness), Moderate (more than one non-critical weakness), Low (one critical flaw with or without non-critical weaknesses), Critically low (more than one critical flaw with or without non-critical weaknesses) through spotting critical and non-critical weaknesses following the recommendation of the AMSTAR 2 developers.^[26]^ And CPGs will be assessed by Appraisal of Guidelines for Research & Evaluation II (AGREE II), which consists of 23 items covering six quality domains, scored with a scale of 1 (totally disagree) to 7 (totally agree) for each.^[28]^ A quality score is calculated for each of the six AGREE II domains. The 6 domain scores are independent and should not be aggregated into a single quality score. Domain scores are calculated by summing up all the scores of the individual items in a domain and by scaling the total as a percentage of the maximum possible score for that domain.

### 2.7 Effect values combination

All the different data types will be converted to odds ratio (OR) and 95% confidence interval (95%CI). Then, the inverse variance method was used to combine the pooled odds ratio (ORP) by using RevMan 5.3, and the risk factors will be included if its ORP is greater than 1 and 95%CI does not include 1.

We will measure statistical heterogeneity using the I^2^ measure. A fixed-effect model will be used if clinical heterogeneity was minimal and I^2^ was less than 50%; otherwise a random-effects model will be adopted due to I^2^ ≥ 75%. We will conduct a sensitivity analysis to identify and remove the literature of significant heterogeneity. We will also perform sensitivity for the included SRs with “critically low” methodological quality one by one, and exclude the SRs which cause great change in overall effect size.

### 2.8 Logistic regression model

Logistic regression model refers to calculate the risk value of risk factors in order to evaluate the contribution of risk factors in the specific diseases and to predict the risk of developing diseases. The risk prediction model will be established based on the natural logarithm transformation value of the ORP. The theoretical prediction model for the risk of pressure injury developing in patients based on logistic regression model is as follows:

$$Logit(P)Ln\left(\frac{P}{1-P}\right)=\alpha +\beta_{1}X_{1}+\beta_{2}X_{2}+\cdots +\beta_{i}X_{i}+\cdots +\beta_{n}X_{n}$$

The risk probability of developing pressure injury will be calculated based on the above model is as follows:

$$P=\frac{e^{\alpha }+\beta_{1}X_{1}+\beta_{2}X_{2}+\cdots +\beta_{i}X_{i}+\cdots +\beta_{n}X_{n}}{1+e^{\alpha +\beta_{1}X_{1}+\beta_{2}X_{2}+\cdots +\beta_{i}X_{i}+\cdots +\beta_{n}X_{n}}}$$

The “X_1_, X_2_, … X_n_” represents the number of risk factors, “β” represents the correspondent natural logarithm transformation value of the ORP ($\beta_{i}=LnORP_{i}$), and “α” will be calculated using the incidence of pressure injury ($\alpha =Ln\left(\frac{P_{0}}{1-P_{0}}\right)$)

### 2.9 Narrative synthesis

Risk factors will be categorized into domains and sub-domains according to the CPG published by the NPIAP, EPUAP, and Pan Pacific Pressure Injury Alliance (PPPIA).^[2]^ Evidence summary tables will be generated for each risk factor domain, with a summary narrative synthesis by sub-domain and domain. In the evidence tables, Grade and Stage are recorded as reported in individual studies. Sankey diagrams may be used to visualize the evidence summary via R software if applicable.

### 2.10 Patient and public involvement

Patients or the public will not be involved in the design, or conduct, or reporting, or dissemination plans of our research.

## 3 Discussion

Our review will provide a synthesis of risk factors and a risk predictive model associated with the development of PIs from systematic reviews and CPGs. In the discussion section in the full report of our study, we are going to include the following subsections:

(1) Summary of main findings;
(2) Interpretation of results;
(3) Strength and limitations;
(4) Comparison of other studies; and
(5) Conclusion.

Although PIs have been given substantial consideration within hospitals and long-term care facilities in recent decades, they remain a significant problem. A summary of the risk factors and a risk predictive model will help to give a direction of prevention interventions for immobile patients and provide the basis for PIs prediction system construction.

## Author contributions

**Conceptualization:** Ke-Lu Yang, Lin Chen, Li-Na Xing, Zong-Hui Song.

**Data curation:** Ke-Lu Yang, Lin Chen, Zong-Hui Song.

**Formal analysis:** Ke-Lu Yang, Lin Chen.

**Funding acquisition:** Zong-Hui Song.

**Investigation:** Zong-Hui Song.

**Methodology:** Ke-Lu Yang, Lin Chen, Ying-Ying Kang, Li-Na Xing, Hai-Ling Li, Peng Cheng, Zong-Hui Song.

**Resources:** Peng Cheng.

**Software:** Ke-Lu Yang, Lin Chen.

**Supervision:** Zong-Hui Song.

**Writing – original draft:** Ke-Lu Yang, Lin Chen, Zong-Hui Song.

**Writing – review & editing:** Ke-Lu Yang, Lin Chen, Zong-Hui Song.
